# Supplementary material for: Hydroxy-Selenomethionine Mitigated Chronic Heat Stress-Induced Porcine Splenic Damage via Activation of Nrf2/Keap1 Signal and Suppression of NFκb and STAT Signal
Source: Int J Mol Sci. 2023 Mar 30;24(7):6461. doi: 10.3390/ijms24076461 (PMC10094443; doi:10.3390/ijms24076461)
Supplement: Supplementary file 1 [file ijms-24-06461-s001.zip › ijms-2282451-supplementary.pdf]

Table S1. Ingredients composition and nutrient levels of the basal diets

Ingredients	Kg per ton
Corn grain	762
Soybean oil	28
Soybean meal	130
Wheat bran	40
Fishmeal (CP 62.5 %)	14
L-Lysine·hydrochloride	4.2
DL-Methionie	1.2
L-Threonine	1.4
L-Tryptophan	0.4
Choline chloride 50%	1.0
Calcium carbonate	8.5
Calcium hydrophosphate	6.5
Sodium chloride	1.8
Premix [a]	1.0
Total	1000
Nutrient levels [b]	
Digestible energy (Kcal/kg)	3400
Crude protein (%)	13.74
Calcium (%)	0.59
STTD Phosphorus (%)	0.28
SID Lysine (%)	0.85
SID Met+cys (%)	0.48
SID Thr (%)	0.52
SID Trp (%)	0.15

[a] Premix provided per kilogram of diet: Fe, 50 mg; Cu, 3.5 mg; Zn, 50 mg; Mn, 2 mg; I, 0.14 mg; vitamin A, 1300 IU; vitamin D_3, 150 IU; vitamin E, 11 mg; vitamin K_3, 0.5 mg; vitamin B_2 2 mg; vitamin B_6, 1 mg; vitamin B_{12}, 5 μg; pantothenic acid, 7 mg; folic acid, 0.3 mg; biotin 0.05 mg.

[b] Nutrient levels were calculated values. The basal diet was a standard experiment diet formulation used in our previous studies (1, 2).

Table S2. Primers used for the q-PCR of the target and reference genes

Protein	Gene	Accession number	Primer sequence (from 5' to 3')
		**Selenoprotein encoding genes**	
DIO1	*DIO1*	AY533206	F: CATGGCCAAGAACCCTCACT

			R: CCAGAAATACTGGGCACTGAAGA
DIO2	*DIO2*	AY533207	F: CGCTGCATCTGGAAGAGCTT<br>R: TGGAATTGGGTGCATCTTCA
DIO3	*DIO3*	AY533208	F: TGAAGTGGAGCTCAACAGTGATG<br>R: TGTCGTCAGACACGCAGATAGG
GPX1	*GPX1*	AF532927	F: GATGCCACTGCCCTCATGA<br>R: TCGAAGTTCCATGCGATGTC
GPX2	*GPX2*	DQ898282	F: AGAATGTGGCCTCGCTCTGA<br>R: GGCATTGCAGCTCGTTGAG
GPX3	*GPX3*	AY368622	F: CCGGTTCCTGTTTTCCAAATT<br>R: TGCACTGCAGGAAGAGTTTGAA
GPX4	*GPX4*	NM_214407	F: TGAGGCAAGACGGAGGTAAACT<br>R: TCCGTAAACCACACTCAGCATATC
GPX6	*GPX6*	NM_001137607	F: GAGCTGAAGCCTTTTGGTGTAGTT<br>R: CTTTGCTGGTTCTTGTTTTCCA
MSRB1	*MSRB1*	EF113597	F: ATCCCTAAAGGCCAAGAATCATC<br>R: GGCCACCAAGCAGTGTTCA
SELENOF	*SELENOF*	EF178474	F: ACAGCCCTGCCAAGCAGAT<br>R: AACAGGGAGGCTGGGTAACAC
SELENOH	*SELENOH*	HM018602	F: TGGTGGAGGAGCTGAAGAAGTAC<br>R: CGTCATAAATGCTCCAACATCAC
SELENOI	*SELENOI*	NM_001244662.1	F: GATGGTGTGGATGGAAAGCAA<br>R: GCCATGGTCAAAGAGTTCTCCTA
SELENOK	*SELENOK*	DQ372075	F: CAGGAAACCCCCCTAGAAGAA<br>R: CTCATCCACCGGCCATTG
SELENOM	*SELENOM*	FJ968780	F: CAGCTGAATCGCCTCAAAGAG<br>R: GAGATGTTTCATGACCAGGTTGTG
SELENON	*SELENON*	EF113595	F: ACCTGGTCCCTGGTGAAAGAG<br>R: AGGCCAGCCAGCTTCTTGT

SELENOO	*SELENOO*	AK236851	F: CTTCCGACCCCAGATGGAT<br>R: GGTTCGACTGTGCCAGCAT
SELENOP	*SELENOP*	EF113596	F: AACCAGAAGCGCCAGACACT<br>R: TGCTGGCATATCTCAGTTCTCAGA
SELENOS	*SELENOS*	AY609646	F: GAGGCAGAGGCACCTGGAT<br>R: CTGCTAAAGCCTCCTGTCGTTT
SELENOT	*SELENOT*	AY609428	F: GGCTTAATAATCGTTGGCAAAGA<br>R: TGGCCCCATTGCCAGATA
SELENOV	*SELENOV*	GQ478346	F: CACTGGTCGCCAATGGATTC<br>R: AGTGGCCAACGGAGAAAGC
SELENOW	*SELENOW*	NM_213977	F: CACCCCTGTCTCCCTGCAT<br>R: GAGCAGGATCACCCCAAACA
SEPHS2	*SEPHS2*	EF033624	F: TGGCTTGATGCACACGTTTAA<br>R: TGCGAGTGTCCCAGAATGC
TXNRD1	*TXNRD1*	AF537300	F: GATTTAACAAGCGGGTCATGGT<br>R: CAACCTACATTCACACACGTTCCT
TXNRD2	*TXNRD2*	GU181287	F: TCTTGAAAGGCGGAAAAGAGAT<br>R: TCGGTCGCCCTCCAGTAG
TXNRD3	*TXNRD3*	BX918808	F: GTGCCCTACGTTTATGCTGTTG<br>R: TCCGAGCCACCAGCTTTG
**Inflammatory response-related genes**			
IL-1β	*IL-1β*	NM_001252429.1	F: TCTGCCCTGTACCCCAACTG<br>R: CCAGGAAGACGGGCTTTTG
IL-6	*IL-6*	NM_213867.1	F: ATGCTTCCAATCTGGGTTCAA<br>R: CACAAGACCGGTGGTGATTCT
IL-8	*IL-8*	NM_214041.1	F: GCAAGAGTAAGTGCAGAACTTCGA<br>R: GGGTGGAAAGGTGTGGAATG
IL-10	*IL-10*	NM_214022.1	F: CAGATGGGCGACTTGTTGCT<br>R: GGCAACCCAGGTAACCCTTAA

TNF-α	*TNF-α*	NM_214015.1	F: CGACTCAGTGCCGAGATCAA<br>R: CCTGCCCAGATTCAGCAAAG
TGF-β	*TGF-β*	JN391525.1	F: AGGACCTGGGCTGGAAGTG<br>R: GGGCCCCAGGCAGAAAT
IFN-β	*IFN-β*	NM_213816.1	F: TCTCTAGCACTGGCTGGAATGA<br>R: CTGCCCATCAAGTTCCACAA
ICAM-1	*ICAM-1*	NM_214214.1	F: GGAGGTGCTGAAATCTCAATGTG<br>R: ACCTTCATGGAGCCTCCTTTG
MCP-1	*MCP-1*	NM_001252429.1	F: GCAAGTGTCCTAAAGAAGCAGTGA<br>R: GCTTGGGTTCTGCACAGATCT
**Heat shock protein**			
HSP70	*HSP70*	NM_001123127.1	F: TTGGGCGCAAGTTTGCA<br>R: GGAAAGGCCAGTGCTTCAAG
**Housekeeping control genes**			
β-ACTIN	*β-ACTIN*	NM_007393.5	F: ACCAGTTCGCCATGGATGAC<br>R: TGCCGGAGCCGTTGTC
GAPDH	*GAPDH*	GU214026.1	F: GGGAAGCCCATCACCATCT<br>R: CGGCCTCACCCCATTTG

The primer used were same as our previous studies (1,2)

Table S3. Primary and secondary antibody information used for the western blot analysis

Primary antibody	Dilution	Source	Identifier	secondary antibody	Dilution	Identifier
HSP70	1:1000	abcam	Ab5439	M[1]	1:10000	ab96879
Nrf2	1:1000	Proteintech	66504-1-lg	M	1:10000	ab96879
Keap1	1:1000	Proteintech	60027-1-lg	M	1:10000	ab96879
HO-1	1:500	Proteintech	27282-1-AP	R[2]	1:20000	ab182016
NQO1	1:1000	Proteintech	11451-1-AP	R	1:20000	ab182016
Caspase3	1:500	Proteintech	19677-1-AP	R	1:20000	ab182016

Bcl-2	1:2000	Proteintech	12789-1-AP	R	1:20000	ab182016
Bax	1:5000	Proteintech	60267-1-lg	M	1:10000	ab96879
p-NFκB	1:1000	Cell signaling technology	#3033	R	1:20000	ab182016
NFκB	1:1000	Cell signaling technology	#6956	R	1:20000	ab182016
p-IκB	1:1000	Cell signaling technology	#6246	R	1:20000	ab182016
IκB	1:1000	Cell signaling technology	#4814	R	1:20000	ab182016
p-p38	1:1000	Cell signaling technology	#4511	R	1:20000	ab182016
p38	1:1000	Cell signaling technology	#8690	R	1:20000	ab182016
p-Stat1	1:1000	Cell signaling technology	#9167	R	1:20000	ab182016
Stat1	1:1000	Cell signaling technology	#14994	R	1:20000	ab182016
p-Stat3	1:1000	Cell signaling technology	#9138	R	1:20000	ab182016
Stat3	1:1000	Cell signaling technology	#9139	R	1:20000	ab182016
GPX1	1:1000	abcam	Ab22604	R	1:20000	ab182016
GPX3	1:500	Santa Cruz Biotechnology	Sc-58361	M	1:10000	ab96879
GPX4	1:500	Zen Bio	381958	R	1:20000	ab182016
SELENOS	1:1000	Proteintech	15591-1-AP	R	1:20000	ab182016
SELENOF	1:1000	Zen Bio	823841	R	1:20000	ab182016
β-actin	1:5000	Millipore	MAB1501	M	1:10000	ab96879

[1] M: Goat Anti-Mouse IgG H&L.

[2] R: Goat Anti-Rabbit IgG H&L

REFERENCE

1. He Y, Liu Y, Tang J, Jia G, Liu G, Tian G, et al. Selenium Exerts Protective Effects against Heat Stress-Induced Barrier Disruption and Inflammation Response in Jejunum of Growing Pigs. *Journal of the science of food and agriculture* (2021) 102(2):496-504. doi: 10.1002/jsfa.11377.
2. Liu Y, Yin S, Tang J, Liu Y, Jia G, Liu G, et al. Hydroxy Selenomethionine Improves Meat Quality through Optimal Skeletal Metabolism and Functions of Selenoproteins of Pigs under Chronic Heat Stress. *Antioxidants (Basel, Switzerland)* (2021) 10(10). doi: 10.3390/antiox10101558.
